# Supplementary material for: Longitudinal EEG power in the first postnatal year differentiates autism outcomes
Source: Nat Commun. 2019 Sep 13;10:4188. doi: 10.1038/s41467-019-12202-9 (PMC6744476; doi:10.1038/s41467-019-12202-9)
Supplement: Supplementary file 1 — Supplementary Information [file 41467_2019_12202_MOESM1_ESM.pdf]

Supplementary Information for:

Longitudinal EEG power in the first postnatal year differentiates autism outcomes by three years

Gabard-Durnam et al.

Supplementary Table 1: Whole-head 3 – 12 months EEG power models

Parameters	ASD vs. HRA-		ASD vs. LRC		HRA- vs. LRC	
	B Coefficient (SE)	p value	B Coefficient (SE)	p value	B Coefficient (SE)	p value
Model intercept	**-14.15 (4.11)**	**0.001**	8.54 (6.29)	0.175	**11.14 (3.23)**	**0.001**
Sex	-	-	1.13 (0.78)	0.149	-	-
Parental Education 1	-	-	-0.16 (1.27)	0.903	**-1.96 (0.94)**	**0.037**
Parental Education 2	-	-	-2.26 (1.2)	0.06	**-2.2 (0.85)**	**0.009**
6-month Intercept						
Delta	-	-	-	-	-9.61 (5.49)	0.08
Theta	-	-	-7.34 (4.22)	0.082	9 (5.91)	0.128
Low Alpha	**10.21 (3.04)**	**0.001**	-	-	**-5.51 (2.78)**	**0.047**
High Alpha	-	-	8.88 (6.65)	0.182	-	-
Beta	-	-	-5.95 (5.47)	0.276	-	-
Gamma	-	-	-	-	-	-
Slope 3-12 months						
Delta	-	-	-	-	-	-
Theta	**9.19 (2.82)**	**0.001**	-	-	**-3.23 (1.08)**	**0.003**
Low Alpha	-	-	-	-	-	-
High Alpha	**-9.65 (3.68)**	**0.009**	-16.68 (14.22)	0.241	-	-
Beta	-	-	13.01 (13.23)	0.326	-	-
Gamma	3.86 (2.14)	0.072	-	-	-	-
Intercept x Slope						
Delta	-	-	-	-	-	-
Theta	-	-	-	-	-	-
Low Alpha	-	-	-	-	-	-
High Alpha	-	-	16.86 (11.74)	0.151	-	-
Beta	-	-	-13.06 (8.69)	0.133	-	-
Gamma	-	-	-	-	-	-

SE = Standard Error; bold values indicate statistically-significant parameters (determined by Student's t-test) within each model at the level of $p < 0.05$

Supplementary Table 2: Whole-head 12 – 24 months EEG power models

Parameters	ASD vs. HRA- B Coefficient (SE)	ASD vs. HRA- p value	ASD vs. LRC B Coefficient (SE)	ASD vs. LRC p value	HRA- vs. LRC B Coefficient (SE)	HRA- vs. LRC p value
Model intercept	-3.59 (6.32)	0.57	**16.46 (7.81)**	**0.035**	**16.06 (4.90)**	**0.001**
Sex	1.62 (0.92)	0.078	1.53 (0.95)	0.11	-	-
Parental Education 1	-	-	0.12 (1.49)	0.93	-	-
Parental Education 2	-	-	**-3.27 (1.43)**	**0.023**	-	-
12-month Intercept						
Delta	**43.19 (18.52)**	**0.020**	10.99 (12.33)	0.37	**-17.75 (8.05)**	**0.027**
Theta	**-49.47 (19.48)**	**0.011**	**-27.39 (13.60)**	**0.044**	8.57 (8.47)	0.31
Low Alpha	**-**	**-**	12.28 (6.65)	0.065	-0.43 (3.98)	0.91
High Alpha	**12.67 (6.11)**	**0.038**	-	-	-	-
Beta	-3.31 (7.50)	0.66	-	-	-	-
Gamma	-3.32 (6.43)	0.61	-7.68 (4.42)	0.08	-	-
Slope 12-24 months						
Delta	**30.84 (11.80)**	**0.009**	-15.85 (13.82)	0.25	**-12.66 (4.93)**	**0.010**
Theta	**-56.95 (21.34)**	**0.008**	-	-	**37.06 (13.69)**	**0.007**
Low Alpha	**-**	**-**	-	-	**-32.40 (13.67)**	**0.018**
High Alpha	**12.67 (6.11)**	**0.038**	**-10.51 (5.35)**	**0.049**	-	-
Beta	-47.52 (30.90)	0.12	**10.85 (5.51)**	**0.049**	-	-
Gamma	21.67 (14.58)	0.14	-6.00 (4.05)	0.14	2.00 (1.42)	0.16
Intercept x Slope						
Delta	-	-	11.20 (8.77)	0.20	-	-
Theta	**20.52 (10.19)**	**0.044**	-	-	-14.60 (7.48)	0.051
Low Alpha	-	-	-	-	15.09 (8.35)	0.071
High Alpha	-	-	-	-	-	-
Beta	29.91 (18.58)	0.11	-	-	-	-
Gamma	-17.44 (10.28)	0.090	-	-	-	-

SE = Standard error; bold values indicate statistically-significant parameters (determined by Student's t-test) within each model at the level of $p < 0.05$

Supplementary Table 3: Whole-head 3 – 36 months EEG power models

Parameters	ASD vs. HRA-		ASD vs. LRC		HRA- vs. LRC	
	B Coefficient (SE)	p value	B Coefficient (SE)	p value	B Coefficient (SE)	p value
Model intercept	**-11.95 (4.83)**	**0.013**	-2.96 (5.71)	0.604	**9.57 (3.15)**	**0.002**
Sex	1.19 (0.75)	0.113	1.17 (0.75)	0.117	-	-
Parental Education 1	0.44 (0.90)	0.622	-0.76 (1.11)	0.495	-0.98 (0.83)	*0.235*
Parental Education 2	-1.41 (0.87)	0.103	**-2.37 (1.04)**	**0.022**	**-1.49 (0.74)**	***0.043***
6-month Intercept						
Delta	3.03 (11.55)	0.793	-	-	-	-
Theta	10.5 (13.63)	0.440	-7.35 (6.25)	0.240	**-4.78 (2.24)**	***0.033***
Low Alpha	**-16.10 (7.52)**	**0.032**	5.69 (6.20)	0.359	-	-
High Alpha	**20.73 (7.89)**	**0.009**	**13.13 (6.29)**	**0.037**	-	-
Beta	-5.66 (3.57)	0.113	-	-	-	-
Gamma	-	-	-7.51 (3.88)	0.053	-1.61 (1.90)	0.400
Slope 3-12 months						
Delta	**114.33 (50.61)**	**0.024**	-	-	-	-
Theta	**-141.71 (56.55)**	**0.012**	-59.40 (31.17)	0.057	**35.39 (11.85)**	**0.003**
Low Alpha	32.20 (20.77)	0.121	**80.58 (29.82)**	**0.007**	-	-
High Alpha	**20.73 (7.89)**	**0.009**	-	-	-	-
Beta	-	-	-	-	-	-
Gamma	-	-	-8.35 (6.92)	0.228	-5.60 (4.21)	0.184
Intercept x Slope						
Delta	**-79.21 (37.73)**	**0.036**	-	-	-	-
Theta	**125.56 (48.44)**	**0.010**	**48.41 (22.39)**	**0.031**	**-29.52 (9.13)**	**0.001**
Low Alpha	**-42.36 (16.58)**	**0.011**	**-65.16 (22.83)**	**0.004**	-	-
High Alpha	-	-	-	-	-	-
Beta	-	-	-	-	-	-
Gamma	-	-	6.62 (6.05)	0.273	4.66 (3.49)	0.181

SE = Standard Error; bold values indicate statistically-significant parameters (determined by Student's t-test) within each model at the level of $p < 0.05$

Supplementary Table 4: Temporal-Parietal 3 – 12 months EEG power models

Parameters	ASD vs. HRA-		ASD vs. LRC		HRA- vs. LRC	
	B Coefficient (SE)	p value	B Coefficient (SE)	p value	B Coefficient (SE)	p value
Model intercept	**-10.75 (3.86)**	**0.005**	2.63 (6.07)	0.66	**9.53 (3.12)**	**0.002**
Sex	-	-	-	-	-	-
Parental Education 1	-	-	-0.32 (1.29)	0.80	**-1.98 (0.93)**	**0.03**
Parental Education 2	-	-	-2.61 (1.31)	0.046	**-2.28 (0.83)**	**0.006**
6-month Intercept						
Delta	-3.04 (4.63)	0.51	-	-	-1.14 (7.94)	0.89
Theta	-	-	-	-	4.15 (7.62)	0.59
Low Alpha	**11.21 (5.15)**	**0.029**	-9.82 (8.89)	0.27	-5.64 (2.94)	0.060
High Alpha	-	-	19.12 (11.00)	0.082	-	-
Beta	-	-	-	-	-	-
Gamma	-	-	**-8.57 (4.24)**	**0.043**	-3.03 (2.36)	0.20
Slope 3-12 months						
Delta	-4.67 (5.23)	0.37	-	-	**23.28 (10.60)**	**0.028**
Theta	-	-	-	-	**-15.62 (7.44)**	**0.036**
Low Alpha	3.96 (2.75)	0.15	**-44.15 (22.16)**	**0.046**	-	-
High Alpha	**-8.60 (3.02)**	**0.004**	**58.52 (25.08)**	**0.020**	-	-
Beta	-	-	-	-	-	-
Gamma	-	-	-4.48 (2.34)	0.055	-4.32 (3.19)	0.18
Intercept x Slope						
Delta	**7.17 (3.43)**	**0.036**	-	-	**-17.07 (7.62)**	**0.025**
Theta	-	-	-	-	10.00 (5.35)	0.062
Low Alpha	-	-	31.33 (16.16)	0.053	-	-
High Alpha	-	-	**-43.83 (19.13)**	**0.022**	-	-
Beta	-	-	-	-	-	-
Gamma	-	-	-	-	3.76 (2.27)	0.097

SE = Standard error; bold values indicate statistically-significant parameters (determined by Student's t-test) within each model at the level of $p < 0.05$

Supplementary Table 5: Temporal-Parietal 12 – 24 months EEG power models

Parameters	ASD vs. HRA-		ASD vs. LRC		HRA- vs. LRC	
	B Coefficient (SE)	p value	B Coefficient (SE)	p value	B Coefficient (SE)	p value
Model intercept	-5.16 (4.02)	0.20	9.31 (6.58)	0.16	**14.02 (4.09)**	**0.0006**
Sex	**1.40 (0.67)**	**0.036**	-	-	-	-
Parental Education 1	-	-	0.73 (1.40)	0.60	-	-
Parental Education 2	-	-	-2.21 (1.29)	0.087	-	-
12-month Intercept						
Delta	**25.36 (10.30)**	**0.014**	**26.57 (13.54)**	**0.050**	-14.60 (7.71)	0.058
Theta	**-23.30 (11.16)**	**0.037**	**-38.17 (13.92)**	**0.006**	4.26 (7.90)	0.59
Low Alpha	-0.61 (5.09)	0.91	-	-	-3.08 (3.74)	0.41
High Alpha	-	-	10.72 (6.15)	0.081	-	-
Beta	-	-	-	-	3.68 (2.39)	0.12
Gamma	-	-	-5.78 (4.42)	0.19	-	-
Slope 12-24 months						
Delta	11.40 (5.83)	0.051	-	-	**-60.50 (21.33)**	**0.005**
Theta	**-43.65 (19.08)**	**0.022**	-	-	**66.60 (21.43)**	**0.002**
Low Alpha	22.77 (15.20)	0.13	-	-	-21.22 (10.94)	0.052
High Alpha	-	-	-32.67 (17.98)	0.069	-	-
Beta	-	-	-	-	2.62 (1.55)	0.091
Gamma	-	-	**28.05 (13.46)**	**0.037**	-	-
Intercept x Slope						
Delta	-	-	-	-	**33.82 (13.81)**	**0.014**
Theta	**23.70 (11.19)**	**0.034**	-	-	**-39.64 (14.05)**	**0.005**
Low Alpha	-15.94 (9.11)	0.080	-	-	9.68 (6.72)	0.15
High Alpha	-	-	22.85 (13.01)	0.079	-	-
Beta	-	-	-	-	-	-
Gamma	-	-	**-17.77 (8.37)**	**0.034**	-	-

SE = Standard error; bold values indicate statistically-significant parameters (determined by Student's t-test) within each model at the level of $p < 0.05$

Supplementary Table 6: Temporal-Parietal 3 – 36 months EEG power models

Parameters	ASD vs. HRA- B Coefficient (SE)	p value	ASD vs. LRC B Coefficient (SE)	p value	HRA- vs. LRC B Coefficient (SE)	p value
Model intercept	**-17.12 (5.91)**	**0.0037**	3.50 (7.53)	0.64	**8.89 (2.42)**	**0.0002**
Sex	1.00 (0.63)	0.11	-	-	-	-
Parental Education 1	-	-	-2.52 (1.61)	0.12	-1.24 (0.83)	0.14
Parental Education 2	-	-	**-4.15 (1.59)**	**0.009**	**-1.62 (0.74)**	**0.029**
6-month Intercept						
Delta	11.22 (9.87)	0.26	20.67 (12.72)	0.10	-	-
Theta	0.44 (9.25)	0.96	-22.76 (13.94)	0.10	**-5.80 (1.70)**	**0.0007**
Low Alpha	-	-	7.47 (7.54)	0.32	-	-
High Alpha	5.87 (7.24)	0.42	-3.24 (10.46)	0.76	-	-
Beta	-1.66 (5.08)	0.74	-	-	-	-
Gamma	-1.82 (5.24)	0.73	-4.63 (4.78)	0.33	-	-
Slope 3-36 months						
Delta	**114.70 (34.00)**	**0.0007**	**218.45 (91.21)**	**0.017**	-	-
Theta	**-142.52 (42.16)**	**0.0007**	**-256.37 (101.69)**	**0.012**	**15.76 (7.78)**	**0.043**
Low Alpha	-	-	**101.54 (43.65)**	**0.020**	-	-
High Alpha	59.62 (38.18)	0.12	-81.04 (46.73)	0.083	-	-
Beta	-50.12 (26.82)	0.062	15.59 (8.60)	0.070	-	-
Gamma	25.79 (15.78)	0.10	**-19.14 (9.52)**	**0.044**	-	-
Intercept x Slope						
Delta	**-80.50 (24.92)**	**0.001**	**-149.48 (63.78)**	**0.019**	-	-
Theta	**120.76 (34.94)**	**0.0005**	**179.84 (71.88)**	**0.012**	**-14.08 (5.53)**	**0.011**
Low Alpha	-	-	**-68.32 (29.42)**	**0.020**	-	-
High Alpha	**-70.23 (33.67)**	**0.037**	58.24 (36.64)	0.11	-	-
Beta	**32.83 (15.17)**	**0.030**	-	-	-	-
Gamma	-16.48 (9.53)	0.084	9.69 (6.66)	0.15	-	-

SE = Standard Error; bold values indicate statistically-significant parameters (determined by Student's t-test) within each model at the level of $p < 0.05$

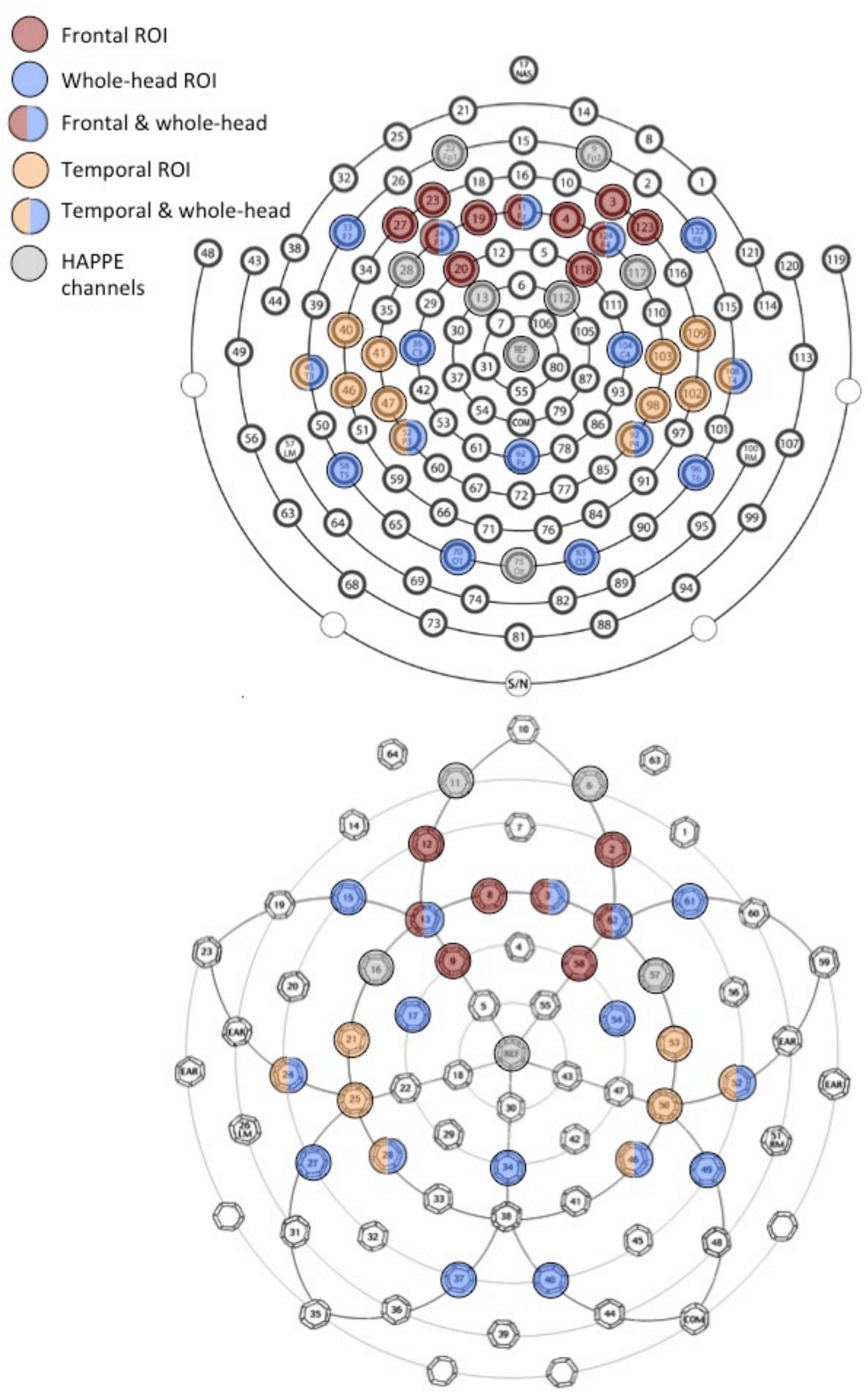

Supplementary Figure 1: EEG Acquisition Net Layouts
The 128-channel EGI HydroCel Geodesic Sensor Net (version 1.0), top panel, and 64-channel EGI Geodesic Sensor Net (version 2.0), bottom panel, used in the study. Each region of interest (ROI) in the main and supplemental analyses is color-coded, with the frontal ROI shown in red, the whole-head ROI shown in blue, and the temporal-parietal (abbreviated to T-P in the figure) ROI shown in orange. HAPPE channels (shown in light grey) refer to the channels selected for preprocessing as part of the HAPPE analysis pipeline.

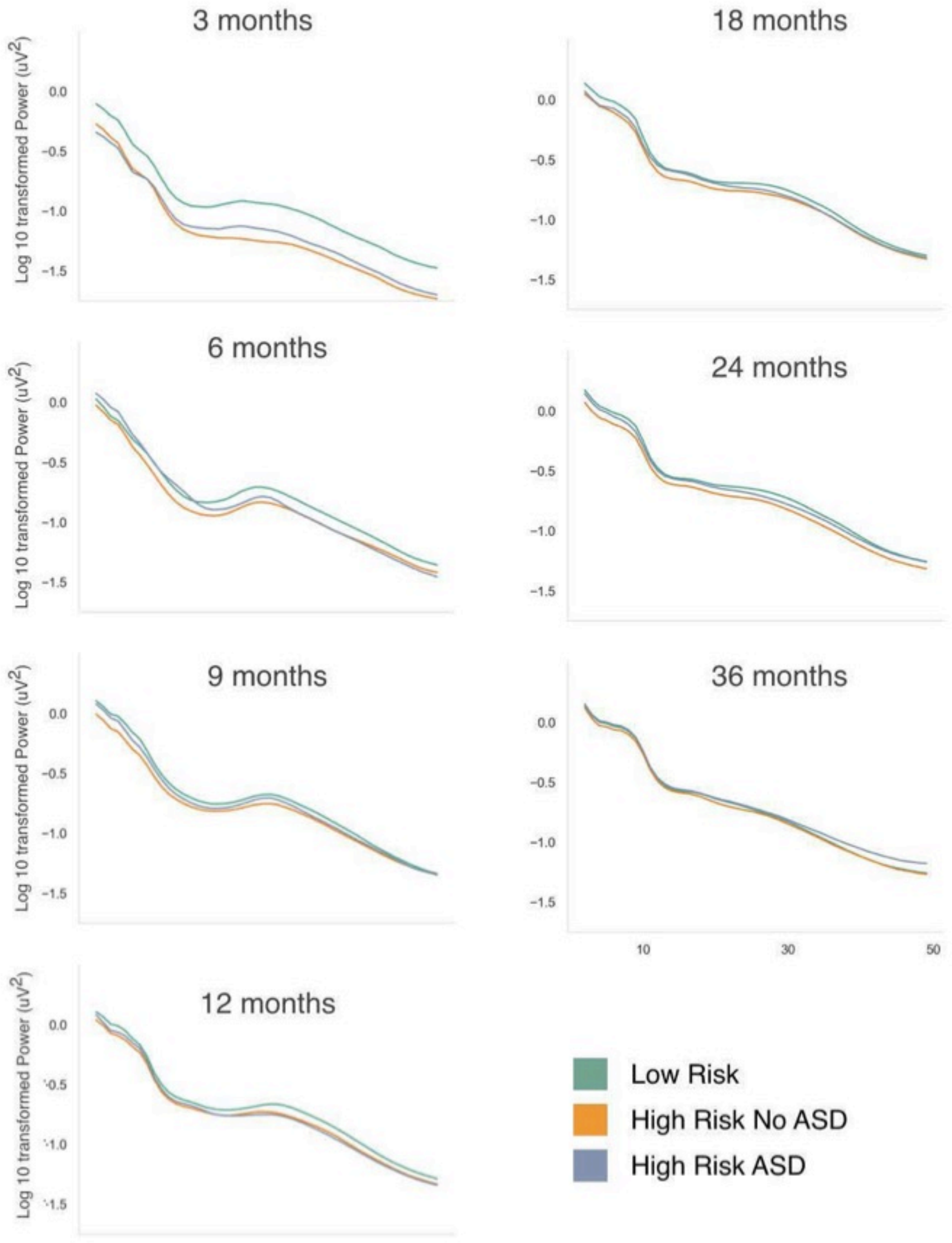

Supplementary Figure 2: EEG Power Distributions Across Age for Frontal Electrodes
The distribution of log-transformed power for all groups, low-risk control (LRC, shown in green), high-risk without ASD (HRA-, shown in orange), and high-risk with ASD (ASD, shown in blue), at each age (in months) for the frontal region of interest. EEG frequency in Hertz is shown on each x-axis (for scale, see 36-month axis). Log (base ten) transformed EEG power in micro-volts squared is provided on each y-axis.
