# Supplementary material for: Longitudinal EEG power in the first postnatal year differentiates autism outcomes
Source: Nat Commun. 2019 Sep 13;10:4188. doi: 10.1038/s41467-019-12202-9 (PMC6744476; doi:10.1038/s41467-019-12202-9)
Supplement: Supplementary file 3 — Reporting Summary [file 41467_2019_12202_MOESM3_ESM.pdf]

nature research

Corresponding author(s): Charles A. Nelson, III

Last updated by author(s): 08/3/2019

Reporting Summary

Nature Research wishes to improve the reproducibility of the work that we publish. This form provides structure for consistency and transparency in reporting. For further information on Nature Research policies, see Authors & Referees and the Editorial Policy Checklist.

Statistics

For all statistical analyses, confirm that the following items are present in the figure legend, table legend, main text, or Methods section.

n/a	Confirmed	
☐	☒	The exact sample size (*n*) for each experimental group/condition, given as a discrete number and unit of measurement
☐	☒	A statement on whether measurements were taken from distinct samples or whether the same sample was measured repeatedly
☐	☒	The statistical test(s) used AND whether they are one- or two-sided<br>*Only common tests should be described solely by name; describe more complex techniques in the Methods section.*
☐	☒	A description of all covariates tested
☐	☒	A description of any assumptions or corrections, such as tests of normality and adjustment for multiple comparisons
☐	☒	A full description of the statistical parameters including central tendency (e.g. means) or other basic estimates (e.g. regression coefficient) AND variation (e.g. standard deviation) or associated estimates of uncertainty (e.g. confidence intervals)
☐	☒	For null hypothesis testing, the test statistic (e.g. *F*, *t*, *r*) with confidence intervals, effect sizes, degrees of freedom and *P* value noted<br>*Give P values as exact values whenever suitable.*
☒	☐	For Bayesian analysis, information on the choice of priors and Markov chain Monte Carlo settings
☒	☐	For hierarchical and complex designs, identification of the appropriate level for tests and full reporting of outcomes
☒	☐	Estimates of effect sizes (e.g. Cohen's *d*, Pearson's *r*), indicating how they were calculated

Our web collection on statistics for biologists contains articles on many of the points above.

Software and code

Policy information about availability of computer code

Data collection	All EEG data were collected using NetStation software (Electrical Geodesics, Inc (EGI), Eugene, OR, USA).
Data analysis	Data were preprocessed using MATLAB software (R2017a), with the open source HAPPE (Gabard-Durnam et al., 2018) and BEAPP (Levin et al., 2018) codes available on GITHUB (HAPPE: https://github.com/lcnhappe/happe; BEAPP: https://github.com/lcnbeapp/beapp). The modeling analyses were carried out in R and SAS software. The code for the modeling is available upon request.

For manuscripts utilizing custom algorithms or software that are central to the research but not yet described in published literature, software must be made available to editors/reviewers. We strongly encourage code deposition in a community repository (e.g. GitHub). See the Nature Research guidelines for submitting code & software for further information.

Data

Policy information about availability of data

All manuscripts must include a data availability statement. This statement should provide the following information, where applicable:

- Accession codes, unique identifiers, or web links for publicly available datasets
- A list of figures that have associated raw data
- A description of any restrictions on data availability

Data Availability
The datasets analysed during the current study are available from the corresponding author on reasonable request.

Field-specific reporting

Please select the one below that is the best fit for your research. If you are not sure, read the appropriate sections before making your selection.

☐ Life sciences ☒ Behavioural & social sciences ☐ Ecological, evolutionary & environmental sciences

For a reference copy of the document with all sections, see nature.com/documents/nr-reporting-summary-flat.pdf

Behavioural & social sciences study design

All studies must disclose on these points even when the disclosure is negative.

Study description

This is a quantitative study.

Research sample

Participants in the present study were part of a prospective, longitudinal investigation across the first 3 years of life of infants at high and low familial risk for ASD. Institutional review board approval was obtained from Boston University and Boston Children's Hospital (# X06-08-0374) prior to the start of the study. Written, informed consent was obtained from a parent or guardian prior to each infant's participation in the study.

Infants were designated high-risk for autism (HRA) if they had at least one older sibling with a community diagnosis of ASD that could not be attributed to a known genetic disorder (e.g. Fragile X syndrome). Infants were designated low-risk for autism controls (LRC) if they had a typically developing older sibling and no first- or second-degree family members with ASD. All infants included in the study had a gestational age of at least 36 weeks, no known prenatal or postnatal medical or neurological complications, and no known genetic disorders (e.g. Fragile X Syndrome, Tuberous Sclerosis Complex).

Final ASD outcomes were determined for all infants using the Autism Diagnostic Observation Schedule (ADOS) in convergence with clinical best estimate. For children meeting criteria on the ADOS, or coming within 3 points of cutoffs, a Licensed Clinical Psychologist reviewed scores and video recordings of concurrent and previous behavioral assessments, and using DSM-V criteria provided a best estimate clinical judgment: typically developing, ASD, or non-spectrum disorder (e.g. ADHD, anxiety, language concerns). By 36 months of age we identified three groups of infants: HRA infants with ASD (ASD), HRA infants without ASD (HRA-), and LRC infants without ASD (LRC). Of the 102 HRA infants contributing data for this study, 4 children had final outcome judgments at 18 months (1 ASD, 3 HRA-), 15 children had final outcome judgments at 24 months (3 ASD, 12 HRA-), and 83 children had final outcome judgments at 36 months (27 ASD, 56 HRA-). The 3 HRA children with HRA- outcomes determined at 18 months of age all had ADOS calibrated severity scores of 1 (minimal to no evidence of ASD), and were not statistical outliers within their HRA- group on any EEG power measure, so they were retained in analyses. Across outcome visits, 31 HRA children met criteria for ASD (ASD outcome group) and 71 were classified as no ASD (HRA- outcome group). None of the 69 LRC infants contributing data met criteria for ASD at outcome visits. EEG data were collected when infants were 3, 6, 9, 12, 18, 24, and 36 months of age. Not all infants contributed adequate EEG data at all timepoints. The sample size of usable EEG for each outcome group at each timepoint and sample sizes for each of the primary analyses run across timepoints are provided in Table 1 of the manuscript and briefly below: Low -risk control (LRC): n = 69 (37 M, 32 F), high-risk no ASD (HRA-): n = 71 (34 M, 37 F), high-risk ASD (ASD): n = 31 (22M, 9 F). For EEG data included in the: 3-12 month analysis and restricted 3-36 month analysis: 64 LRC, 51 HRA-, 23 ASD; 12-24 month analysis: 54 LRC, 48 HRA-, 25 ASD.

Sampling strategy

The sample was a convenience sample. To ensure enough EEGs would be collected from high-risk infants who would go on to have ASD to theoretically meet normality assumptions for statistical tests (~ n = 20), assuming a conversion rate of 20-30% for the high-risk infants, around 100 high-risk infants were recruited to achieve the ~30 sample size for the ASD group.

Data collection

Baseline, resting EEG data were acquired while the infants were seated on their caregivers' laps in a dimly lit, sound-attenuated, electrically shielded room. Continuous EEG was recorded for between 2 and 5 minutes while a research assistant blew bubbles and/or showed toys to keep the infants still. EEG data were collected with Geodesic Sensor Nets, using a 0.1 Hz high-pass analog (i.e. hardware) filter and online re-referencing to the vertex (channel Cz) through NetStation software (Electrical Geodesics, Inc (EGI), Eugene, OR, USA). The researcher was not blind to risk-group status, although for all visits everyone was blind to eventual outcome status as ASD diagnosis had not been identified until after the last EEG collection.

Timing

Data collection started in 2007 and ended in 2015 for the study.

Data exclusions

A priori data exclusion: HAPPE's data quality metrics were used to identify and reject problematic EEG files post-automated processing using recommended thresholds. EEGs were rejected if they had fewer than 20 post-processed good segments (40 total seconds of EEG), or were more than 3 standard deviations (SD) from the mean on the following HAPPE metrics: percent good channels (3 SD: < 82%), mean retained artifact probability (3 SD: > 0.3), median retained artifact probability (3 SD: > 0.35), percent of independent components rejected (3 SD: >84%), and percent of signal variance retained after artifact removal (3 SD: <32%). EEGs with a mean power greater or less than two SD from their outcome group for each frequency band were visually reviewed blind to outcome group. This lead to 25 additional EEGs to be rejected (9 LRC EEGs, 11 HRA- EEGs, and 5 ASD EEGs). HAPPE quality metrics and visual inspection rejection rates did not significantly differ between any pair of outcome groups (all p > 0.1, Table 1 in manuscript). No further data exclusions were made.

Non-participation

A total of 255 participants were enrolled in the study. After enrollment, 19 participants were excluded from data analysis as they no longer met criteria after additional information was acquired: In utero drug exposure (N=1), participant found to be half-sibling of proband (N=4), LR participants with family history of ASD (N=5), and proband of LR participant met criteria on ADOS (N=9).

In addition, several participants were excluded during the study for the following :hearing impairment (N=1), seizures (N=1), genetic finding associated with developmental delays (N=1).Of the remaining 233 participants, infants were included in the present analyses if they (1) were assessed for ASD diagnosis at 18, 24, or 36 months, (174 participants) and (2) had high-quality spontaneous EEG for at least 2 timepoints within 3-12 months or 12-24 months ranges (see above for the EEG data exclusion criteria) (171 of the 174 infants).

	Therefore, 171 infants fulfilled these criteria and were included in analyses.
Randomization	Participants were allocated into groups depending on their family history of ASD status. Infants were designated high-risk for autism (HRA) if they had at least one older sibling with a community diagnosis of ASD that could not be attributed to a known genetic disorder (e.g. Fragile X syndrome). Infants were designated low-risk for autism controls (LRC) if they had a typically developing older sibling and no first- or second-degree family members with ASD. All infants included in the study had a gestational age of at least 36 weeks, no known prenatal or postnatal medical or neurological complications, and no known genetic disorders (e.g. Fragile X Syndrome, Tuberous Sclerosis Complex). The ASD group had significantly more male children than the HRA- group (Pearson X2 (1, 102) = 4.64, p = 0.031. The LRC group had higher mean parental education level than both the ASD group (Pearson X2 (2, 87) = 12.65, p = 0.002) and the HRA- group (Pearson X2 (2, 127) = 6.62, p = 0.036). Accordingly, sex and parental education parameters were included as potential covariates during model selection to ensure the EEG parameters explained the variance in outcome status unrelated to these demographic parameters. Supplemental analyses were also performed without the sex and education parameters as potential covariates, and results were highly consistent with the reported results.

Reporting for specific materials, systems and methods

We require information from authors about some types of materials, experimental systems and methods used in many studies. Here, indicate whether each material, system or method listed is relevant to your study. If you are not sure if a list item applies to your research, read the appropriate section before selecting a response.

Materials & experimental systems

n/a	Involved in the study
☒	☐ Antibodies
☒	☐ Eukaryotic cell lines
☒	☐ Palaeontology
☒	☐ Animals and other organisms
☐	☒ Human research participants
☒	☐ Clinical data

Methods

n/a	Involved in the study
☒	☐ ChIP-seq
☒	☐ Flow cytometry
☒	☐ MRI-based neuroimaging

Human research participants

Policy information about studies involving human research participants

Population characteristics	see above
Recruitment	Participants were recruited through the Boston Children's Hospital internal subject database (where families can opt in after the birth of their child), through Autism outreach activities in the local area, and through doctor referrals at Boston Children's Hospital (if older siblings received a diagnosis of ASD).
Ethics oversight	Ethics approval was provided by the Institutional Review Board (IRB) at Boston Children's Hospital and the IRB at Boston University. Approved study protocol (# X06-08-0374).

Note that full information on the approval of the study protocol must also be provided in the manuscript.
